# Genome-Wide Identification and Expression Profiling of CONSTANS-Like Genes in Pepper (*Capsicum annuum*): Gaining an Insight to Their Phylogenetic Evolution and Stress-Specific Roles

**DOI:** 10.3389/fpls.2022.828209

**Published:** 2022-02-17

**Authors:** Zhinan Huang, Xueying Bai, Weike Duan, Boqing Chen, Guodong Chen, Binghua Xu, Rui Cheng, Jizhong Wang

**Affiliations:** ^1^College of Life Science and Food Engineering, Huaiyin Institute of Technology, Huai’an, China; ^2^Huai’an Key Laboratory for Facility Vegetables, Huaiyin Institute of Agricultural Sciences of Xuhuai Region in Jiangsu, Huai’an, China

**Keywords:** COL, phylogenetic relationship, expression pattern, abiotic stress, *Capsicum annuum*

## Abstract

*CONSTANS-like* (*COL*) genes play important regulatory roles in multiple growth and development processes of plants but have rarely been studied in *Capsicum annuum*. This study explored the evolutionary relationship and expression patterns of *COL* genes from *C. annuum*. A total of 10 *COL* genes were identified in the genome of the cultivated pepper *Zunla-1* and were named *CaCOL01*–*10*. These genes were unequally distributed among five chromosomes and could be divided into three groups based on differences in gene structure characteristics. During evolutionary history, duplications and retentions were divergent among different groups of *COL* genes. Tandem duplication caused amplification of group I genes. Genetic distance among *COL* genes was the largest in group III, suggesting that group III genes undergo more relaxed selection pressure compared with the other groups. Expression patterns of *CaCOLs* in tissues were significantly different, with *CaCOL08* exhibiting the highest expression in stem and leaf. Some *COL* orthologous genes showed markedly different expression patterns in pepper compared with tomato, such as COL_1 orthologs, which may be involved in fruit development in pepper. In addition, *CaCOLs* participated in the regulation of abiotic stresses to varying degrees. Five *CaCOL* genes were induced by cold, and *CaCOL02* and *CaCOL03* were specifically upregulated by cold and downregulated by heat. This study provides a theoretical basis for the in-depth understanding of the functions of *COL* genes in pepper and their molecular mechanisms involved in growth and development and responses to abiotic stresses.

## Introduction

*CONSTANS* (*CO)*, a crucial gene in the photoperiod flowering induction pathway of plants, is located in the circadian clock output pathway and promotes the flowering of plants by activating expression of the downstream floral gene *FLOWERING LOCUS T* (*FT*) (Putterill et al., 1995). *CO* was subsequently found to belong to a class of genes called the *CONSTANS-like* (*COL*) gene family (Griffiths et al., 2003). The *COL* gene family is widely distributed and the members vary greatly among different plant species. For example, 17 *COL* genes were identified in *Arabidopsis thaliana* (Griffiths et al., 2003), 9 in barley (Griffiths et al., 2003), 16 in rice (Griffiths et al., 2003), 26 in soybean (Wu et al., 2014), 42 in cotton (Cai et al., 2017), 19 in maize (Song et al., 2018), 20 in radish (Hu et al., 2018), 13 in tomato (Yang et al., 2020), and 22 in sunflower (Niu et al., 2022). *COL* genes are characterized based on two conservative domains. The first is the CONSTANS, CONSTANS-LIKE, TOC1 (CCT) domain at the C-terminus, which comprises 43 amino acids and conservatively plays a role in promoter elements binding, transcription regulation, and nuclear localization. The N-terminus of *COL* genes generally contains one to two B-box type zinc finger structures that can interact with ubiquitin ligase, receptor proteins, and transcriptional regulators (Robson et al., 2001; Valverde et al., 2004). According to the number and conservation of B-boxes, the *A. thaliana COL* gene family can be divided into three groups (Griffiths et al., 2003). Furthermore, in most other studied plants, the *COL* gene family clusters into three groups with strong bootstrap values. Both group I and group III genes stably contain two B-box domains in terrestrial plants before *Amborella trichopoda*. After the angiosperm whole-genome duplication (WGD) events, the first B-box domain in group I and the second B-box domain in group III were lost in some plant species (Song et al., 2015). Differentiation of the B-box domain has a crucial role in the phyletic evolution of *COL* genes (Song et al., 2015; Niu et al., 2022).

As transcription factors, *CO* was the first cloned in *A. thaliana*, regulating the flowering time in the photoperiod pathway (Putterill et al., 1995; Li and Xu, 2017). The function of *CO* may be conserved across the angiosperms. For example, *CrCO* (of *Chlamydomonas reinhardtii*) was overexpressed in *A. thaliana* to make flowering in advance (Tenorio et al., 2003), and *PtCO* was found to promote the early flowering of plants (Bohlenius et al., 2006). However, during evolutionary history, *COL* genes often exist in plants as multiple copies, displaying apparent functional differentiation in different plants. Moreover, not all the genes of this family are photoperiod related; several reports have demonstrated that *COL* genes are associated with plant development and even abiotic stress responses. *CrCO* may also participate in cell division and regulation of starch synthesis (Tenorio et al., 2003). In *Populus*, *PtCO* was involved in plant growth and bud differentiation (Bohlenius et al., 2006). *AtCOL3* promoted the development of lateral roots and shoot branches (Datta et al., 2006). *VvCOL1* was involved in regulating the maintenance and induction of bud dormancy in grape (Almada et al., 2009). *MaCOL1*, a transcriptional activator in banana, was reported to be associated with fruit ripening and abiotic stress tolerance (Chen et al., 2012). *AtCOL4* was proven to positively regulate tolerance to abiotic stresses (acid, salt, and osmotic stress) in the plant (Min et al., 2015). In rice, *Ghd2* participated in regulating leaf senescence and drought tolerance (Liu J. et al., 2016). *OsCOL9* was reported to regulate disease resistance dependent on ethylene and salicylic acid (SA) signaling pathways (Liu H. et al., 2016). *BnCOL2* in *Brassica napus* responded to drought stress (Liu et al., 2020), while *HaCOL3*, *HaCOL6*, and *HaCOL19* were induced by cadmium (Cd), heat, and drought stresses in sunflower (Niu et al., 2022). In adapting to adverse environments, *COL* genes have developed essential new functions in response to abiotic stresses.

CONSTANS-like genes have been studied in many plants, but there are no systematic reports of *COLs* in *Capsicum annuum*, a vital vegetable crop. Publication of the whole-genome sequence and high-quality physical map of *C. annuum* has laid the foundation for phylogenetic evolution and functional research of *COL* genes (Kim et al., 2014; Qin et al., 2014). Systematically analyzing conserved domains, evolutionary relationships, and the functional expression of *CaCOL* genes would provide theoretical support for further investigation of the molecular mechanism of *CaCOL* genes and vegetable-breeding research.

## Results

### Members of the *CaCOL* Gene Family

Based on genome-wide identification and validation, all 10 *COL* gene family members were identified in the *C. annuum* cv. *Zunla-1* genome and were named *CaCOL01-CaCOL10* in order of chromosomal location (Table 1). Simultaneously, nine *COLs* were identified in *C. annuum* cv. *CM334* (Supplementary Table 1). The lengths of the 10 *COL* genes in the “*Zunla-1*” genome varied, with a maximum of 1410 bp (*CaCOL09*) and a minimum of 1,032 bp (*CaCOL01*). The molecular weights of CaCOLs were predicted to be between 37.93∼51.67 kD, and the isoelectric points were concentrated in 4.85∼7.07 (Table 1). Comparative analysis indicated that *CaCOL* genes and *AtCOL* genes had high sequence similarity (*E*-value < 5E-44).

**TABLE 1:** The information of COL gene family in pepper *Zunla-1*.

Gene name	Gene accession No.	Size/aa	Length/bp	Chromosome location	MW/kD	pI	*Arabidopsis* homologous	*E*-value
*CaCOL01*	Capana01g004030	343	1,032	Chr01:278243944..278245872	37.93	5.44	AtCOL4	5.00E-94
*CaCOL02*	Capana02g003199	404	1,215	Chr02:157107846..157110095	44.56	5.42	AtCOL2	2.00E-97
*CaCOL03*	Capana02g003200	398	1,197	Chr02:157118313..157120062	43.89	5.23	AtCOL2	5.00E-44
*CaCOL04*	Capana02g003201	407	1,224	Chr02:157124787..157126407	45.39	5.27	AtCOL2	7.00E-84
*CaCOL05*	Capana03g000377	382	1,149	Chr03:5273465..5275489	43.46	5.52	AtCOL6	3.00E-90
*CaCOL06*	Capana03g003558	387	1,164	Chr03:228734754..228738294	43.34	5.45	AtCOL13	7.00E-83
*CaCOL07*	Capana07g000030	384	1,155	Chr07:1563552..1565426	42.54	6.99	AtCOL5	1.00E-89
*CaCOL08*	Capana12g000414	356	1,071	Chr12:8179392..8181909	39.43	5.24	AtCOL4	2.00E-97
*CaCOL09*	Capana00g001486	469	1,410	Chr00:399610115..399614902	51.67	7.07	AtCOL14	1.00E-97
*CaCOL10*	Capana00g004489	426	1,281	Chr00:649683777..649686080	46.86	4.85	AtCOL12	7.00E-103

*MW, molecular weight; pI, isoelectric point.*

### Phylogenetic and Structure Characterization of CaCOLs

**FIGURE 1:**
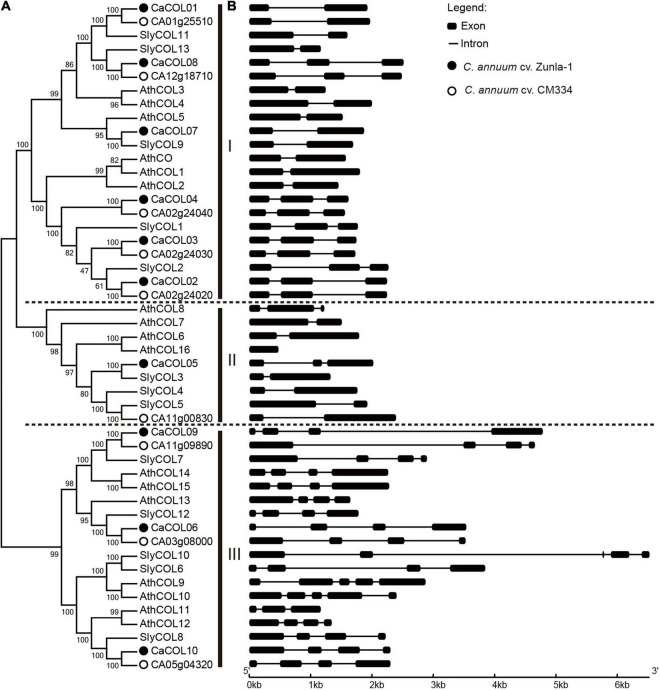
Phylogenetic analysis **(A)** and gene structures **(B)** of COL homologs in *Capsicum annuum*, *Solanum lycopersicum*, and *Arabidopsis thaliana.* The phylogenetic relationship of COLs categorized them into three groups (I, II, and III). COLs of *C. annuum Zunla-1* and CM334 are indicated by black balls and hollow circles, respectively. Exons and introns are represented by black boxes and black lines, respectively.

To understand the phylogenetic evolution of CaCOLs, the COL sequences of *A. thaliana*, tomato, and *C. annuum* cv. *Zunla-1* and *CM334* were selected for analysis. The COLs were divided into three groups (I, II, and III). In “*Zunla-1*,” group I contained six COLs (*CaCOL01*, *CaCOL02*, *CaCOL03*, *CaCOL04*, *CaCOL07*, and *CaCOL08*), which was similar to the number of COLs in group I of *A. thaliana* and tomato. Group II of “*Zunla-1*” contained only one gene (*CaCOL05*), while group III contained three COLs (*CaCOL06*, *CaCOL09*, and *CaCOL10*) (Figure 1A). In the “*CM334*” genome, there were five COLs in group I, with one missing compared to “*Zunla-1*,” groups II and III contained the same number of COLs as “*Zunla-1*.” There were eight pairs of homologous COLs in the two varieties of *C. annuum* (Figure 1A).

There was apparent differentiation in gene structure characteristics among the three subgroups of COLs, but a relatively similar gene structure was present within the groups (Figure 1B). The number of exons in groups I and II was approximately two to three but reached approximately five in group III. In addition, the intron lengths of COLs in groups I and II were similar. *CaCOL09* and *SlyCOL10*, contained longer introns than the other genes, exceeding 4 kb. Such phenomenon of intron-exon structural alienation may be due to differentiation of gene function during evolution.

**FIGURE 2:**
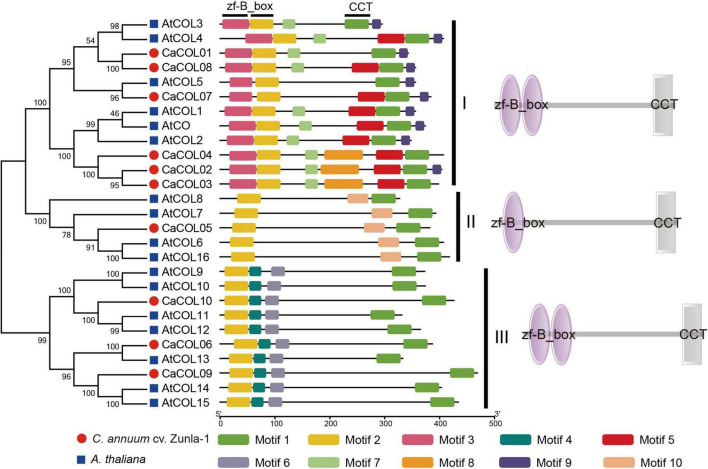
Analysis of conserved domains of COL proteins in *Capsicum annuum* and *Arabidopsis thaliana.* CaCOLs and AtCOLs are indicated by red balls and blue boxes, respectively. B-box and CCT domains were annotated by the SMART database.

Through analysis of the conserved domains of *A. thaliana* and *C. annuum* cv. *Zunla-1* COL proteins (Figure 2), 10 conserved motifs were identified and annotated. Each COL protein contained Motif 1 and 2, indicating that the CCT domain and the B-box domain were the essential functional domains. However, in the three subgroups, there was significant differentiation of B-box domains. Groups I and III both contained two B-boxes, but only one B-box domain was conserved, with the first B-box in group I and the second B-box in group III exhibiting markedly different structures. There was only one B-box domain in group II (Figure 2). Among the three subgroups, group I had the most complex structure and contained seven types of motifs, group II had the simplest motif type, with only three types, and group III contained four types of motifs. COL homologous proteins of *A. thaliana* and *C. annuum* had similar conserved domains, indicating that they may have similar functions (Figure 2).

### *Cis*-Elements Analysis in the Promoters of *CaCOL* Genes

**FIGURE 3:**
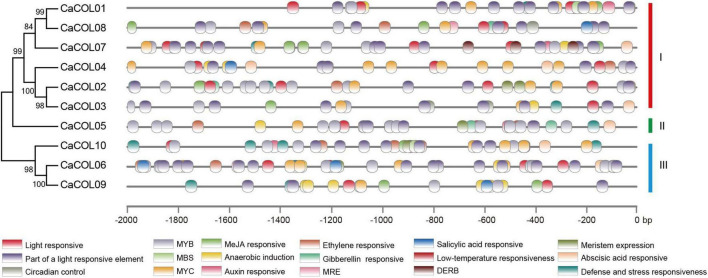
Predicted *cis*-elements in the promoter regions of *CaCOL* genes. Promoter sequences (-2000 bp) of *CaCOL* genes were analyzed. The elements are identified as different colored boxes.

The *cis*-elements in the upstream 2-kb regions of the *CaCOL* gene sequences were analyzed to predict the functions of *CaCOLs*. The promoter regions of *CaCOL* genes contained different types and numbers of *cis*-elements (Figure 3). All 10 genes contained light response *cis*-elements, the stress response *cis*-element (MYB), and the cold resistance *cis*-element recognition site (MYC). In addition, two of the *CaCOL* genes contained the drought response *cis*-element (MBS), and nine of the genes contained the abscisic acid response *cis*-element (ABRE). The *CaCOL* genes also included a variety of hormone response *cis*-elements. These findings indicated that *CaCOLs* might participate in plant growth, development, and resistance to adversity stresses.

### Chromosomal Location and Homologous Genes of *CaCOL* Genes

**FIGURE 4:**
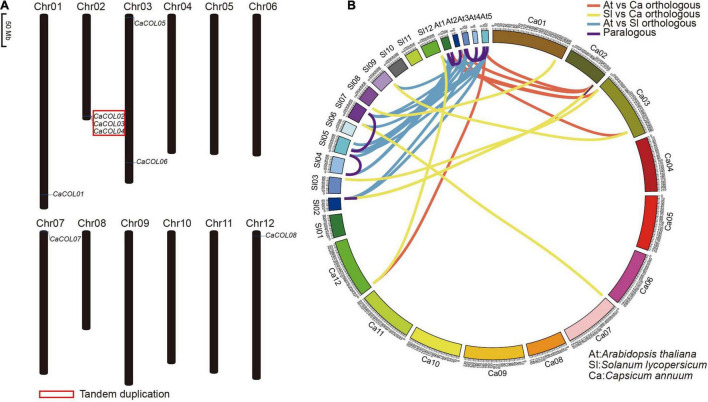
Chromosomal localization of *CaCOL* genes **(A)** and the relationship of *COL* homologous genes among *Capsicum annuum*, *Solanum lycopersicum*, and *Arabidopsis thaliana* **(B)**. The red box indicates tandem duplicated genes. Lines showing orthologous gene pairs between *A. thaliana* and *C. annuum* are red; those between *S. lycopersicum* and *C. annuum* are yellow; and those between *A. thaliana* and *S. lycopersicum* are blue. Lines showing paralogous gene pairs are pink.

Chromosome location analysis showed that eight of the ten *CaCOL* genes were anchored on five chromosomes of *C. annuum*—Chr01, Chr02, Chr03, Chr07, and Chr12 (Figure 4A). Three tandem duplicated genes were found on Chr02 (Figure 4A).

*COL* homologous analysis identified six orthologous gene pairs between *A. thaliana* and *C. annuum*, eight pairs between *A. thaliana* and tomato, and 13 pairs between *C. annuum* and tomato. In addition, only three paralogous gene pairs were found in *C. annuum* and tomato, while seven were identified in *A. thaliana* (Figure 4B and Supplementary Table 2).

### Evolution Pattern Analysis of CONSTANS-Like Genes

**FIGURE 5:**
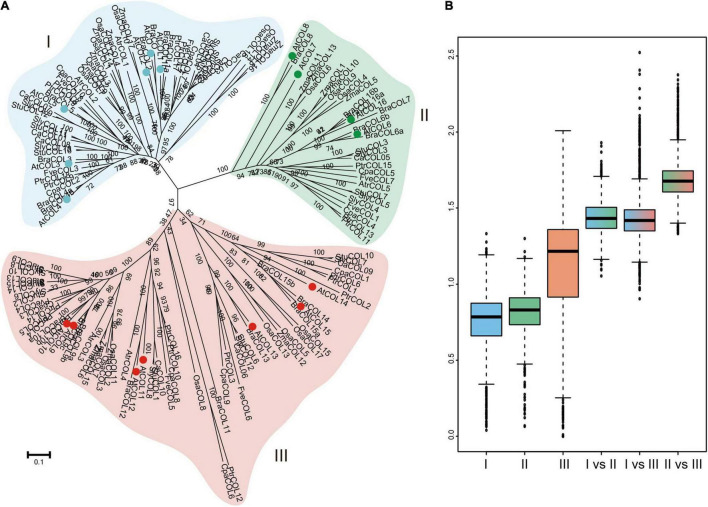
Phylogenetic relationship of *COL* gene family members in 12 selected plant species **(A)** and nucleotide distance of group I, II, and III *COL* genes **(B)**. Group I, II and III of AtCOLs are indicated by blue, green and red balls, respectively.

CONSTANS-like gene family members of 12 plant species (*Arabidopsis thaliana*, *Brassica rapa*, *Carica papaya*, *Populus trichocarpa*, *Fragaria vesca*, *Vitis vinifera*, *Solanum tuberosum*, *Solanum lycopersicum*, *C. annuum* cv. *Zunla-1, Zea mays*, *Oryza sativa*, and *Amborella trichopoda*) were selected to reconstruct a phylogenetic tree (Supplementary Table 3) and further explore the evolutionary history of COLs. In the chosen angiosperms, *A. trichopoda* is one of the species known to exist for the longest time among existing angiosperms without experiencing ancient hexaploid (γ) events, while *C. papaya*, *P. trichocarpa*, and *V. vinifera* did not undergo α and β duplications, and Solanaceae crops have experienced unique whole-genome triplication (WGT) events. All *COL* genes were divided into three subgroups: groups I, II, and III (Figure 5A). Genetic distance was analyzed to further determine the relationship among the three subgroups (Figure 5B). The box plot revealed that genetic distances of the *COL* genes were the smallest in group I and the largest in group III, indicating that the degree of sequence divergence of group I was the lowest. The genetic distance of group II versus group III was higher than those of group I versus group II and group I versus group III, indicating that groups II and III may be differentiated from group I during the evolutionary process. The genetic distance was bigger among the three groups than within the three groups, confirming that the COL gene family was divided into three groups.

**FIGURE 6:**
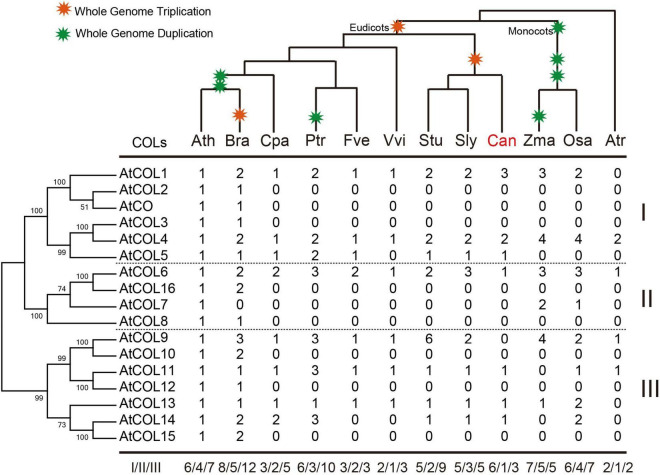
Copy number variation of the COL family in 12 selected plant species. The phylogenetic tree of AtCOLs is shown on the left, and the species tree is shown at the top. Whole-genome duplication and triplication events are indicated on the tree branches according to the Plant Genome Duplication Database. Numbers are copy numbers of each gene in *Arabidopsis thaliana* (Ath), *Brassica rapa* (Bra), *Carica papaya* (Cpa), *Populus trichocarpa* (Ptr), *Fragaria vesca* (Fve), *Vitis vinifera* (Vvi), *Solanum tuberosum* (Stu), *Solanum lycopersicum* (Sly), *Capsicum annuum* (Can), *Zea mays* (Zma), *Oryza sativa* (Osa), and *Amborella trichopoda* (Atr). Statistics of copy number variation of the *COL* genes are based on the phylogenetic relationships.

Copy number variation of the COL family in the 12 selected plant species was subsequently determined (Figure 6). The COLs in *A. trichopoda* had already been divided into three subgroups (Figure 6). In *A. thaliana* and *B. rapa*, the cruciferous plants, groups I and III had the most apparent gene amplification (Figure 6). The number of *COL* genes in *Z. mays* and *O. sativa* was the same, but group III genes of *O. sativa* had been significantly amplified and differentiated, indicating that these genes may play a regulatory role in the growth of *O. sativa* (Figure 6). In the three Solanaceae crops, the copy number of *COL* genes was significantly different; *S. tuberosum* contained the most, while *C. annuum* had the least. Moreover, in *C. annuum*, only one group II gene was retained (Figure 6).

### Expression Divergence of the *CaCOL* Genes in Different Tissues

**FIGURE 7:**
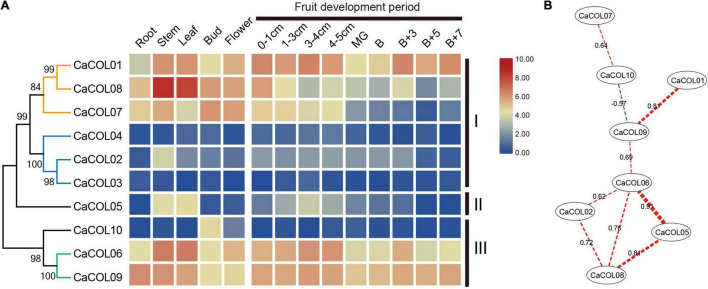
Expression patterns **(A)** and co-expression networks **(B)** of *CaCOL* genes in different tissues and at different stages of the fruit development process. Co-expression networks of *CaCOL* genes were established based on the PCCs of these gene pairs using transcriptome data, which involved eight nodes and nine regulatory edges.

To investigate the divergence of *COL* homologs, the expression patterns of *CaCOL* genes were analyzed based on the transcriptome data of different tissues and fruit development stages of *C. annuum* cv. *Zunla-1*. The expression patterns of *CaCOL* genes showed tissue specificity and a high degree of similarity between genes from the same clade of the phylogenetic tree (Figure 7A). In group I, the genes were clustered into two clades. The first clade included *CaCOL01*, *CaCOL07*, and *CaCOL08*, All three of these genes exhibited high expression in the primary tissues, with *CaCOL08* showing the highest expression of all *CaCOL* genes in the stem and leaf, while *CaCOL07* was expressed at a low level once the fruit had turned color. The second clade of group I comprised *CaCOL02*, *CaCOL03*, and *CaCOL04*, and these genes were either not expressed or were expressed at low levels in most of the investigated tissues, except for *CaCOL02*, which was only expressed at moderate levels in the stem. The sole gene of group II—*CaCOL05—*was expressed at high levels in the stem and leaf but exhibited low or no expression in other tissues. In group III, *CaCOL06*, *CaCOL09*, and *CaCOL10* showed two distinct expression patterns. *CaCOL10* was barely expressed in all tissues except in the stem, while *COL06* and *CaCOL09* were expressed at high or moderate levels in all tissues tested. These findings indicate that *CaCOL* genes play a crucial role in plant growth and undergo functional differentiation (Figure 7A).

To further understand the correlation between *CaCOL* genes and their homologous genes, correlation between the expression trends of *CaCOL* genes was explored by calculating Pearson Correlation Coefficient (PCC) values and then constructing a co-regulatory network by screening significantly co-expressed gene pairs (Figure 7B). *CaCOL06* and *CaCOL09* were at the central node of the co-expression network, and *CaCOL06* had a strong co-expression relationship with four genes simultaneously, indicating that this gene may be at the central node position in the *C. annuum* growth and development regulatory network (Figure 7B).

Subsequently, transcriptome data of different tissues and developmental stages of *C. annuum* cv. *CM334* and tomato were analyzed (Supplementary Figures 1, 2), and the expression patterns of seven groups of *COL* orthologous genes were compared (Supplementary Figure 3). The expression patterns of *COL* orthologous genes were both similar and opposite in pepper and tomato. Four of the seven groups of *COL* orthologous genes showed almost identical expression patterns, namely COL_2, COL_5, COL_6, and COL_7. Orthologous genes in the COL_2 group were highly expressed in leaf tissues of both tomato and pepper, and at low levels during fruit development, especially at breaker stage. In the COL_5 group, the orthologous genes were expressed highly in bud tissues. Both COL_6 and COL_7 orthologous genes were expressed in all tissues tested, but expression levels were higher in pepper compared with tomato (Supplementary Figure 3). The other three groups of orthologous genes (COL_1, COL_3, and COL_4) showed different expression patterns in the two plant species. COL_1 genes were highly expressed in pepper fruit but not in tomato fruit at the breaker stage, while COL_3 genes were the opposite, with very low expression or almost no expression in pepper fruit and higher expression in tomato fruit. COL_4 genes were barely expressed in all tissues of pepper but were expressed in tomato throughout fruit development (Supplementary Figure 3). The expression patterns of most genes were consistent in both pepper varieties, with only the COL_2 genes being expressed in the “*Zunla-1*” stem at higher levels than in “*CM334*” (Supplementary Figure 3).

### Diurnal Rhythm Expression Patterns of *CaCOL* Genes

**FIGURE 8:**
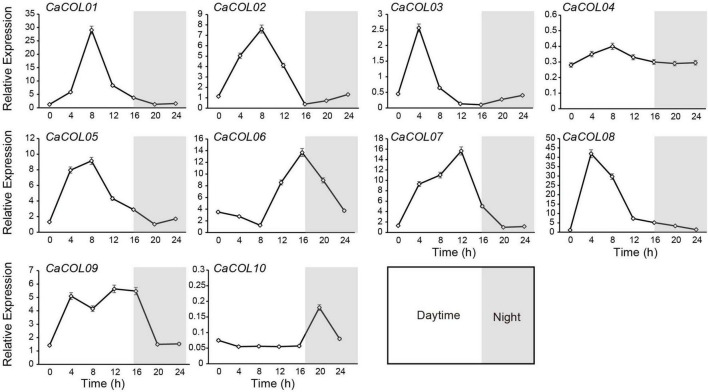
Effect of light on the expression patterns of *COLs* in pepper leaves. Line charts indicate transcript levels of *COL* genes. For each sample, transcript levels were normalized with those of actin (control). Data are the mean values ± standard deviation (SD) of three individual experiments (*n* = 3).

CONSTANS-like genes are reported to exhibit prominent circadian rhythm characteristics. Therefore, the expression patterns of 10 *CaCOLs* during 24 h were studied under long-day (LD) conditions. All genes, except *CaCOL04* and *CaCOL10*, could be induced by light conditions (Figure 8). During the 24 h cycle, the expression of *CaCOL01*, *CaCOL02*, *CaCOL03*, *CaCOL05*, and *CaCOL08* peaked and then decreased at 4 or 8 h after dawn and did not change significantly in the dark (Figure 8). *CaCOL07* reached a peak at 12 h, while *CaCOL06* peaked at 16 h, and *CaCOL09* maintained a high expression level from 4 to 16 h (Figure 8).

### Expression Patterns of CaCOLs Under Abiotic Stresses

**FIGURE 9:**
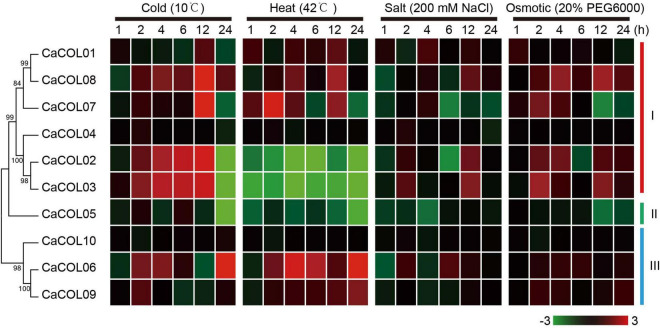
Expression of *CaCOL* genes under cold, heat, salt, and osmotic stress. The bar at the bottom of each heat map represents the relative expression values.

The expression patterns of the 10 *CaCOL* genes under four abiotic stresses (cold, heat, salt, and osmotic) were analyzed by qRT-PCR (Figure 9). Under cold treatment, five *CaCOL* genes were induced, among which the expression of *CaCOL02*, *CaCOL03*, *CaCOL07*, and *CaCOL8* peaked at 12 h after treatment and then decreased, while *CaCOL06* expression peaked at 24 h. Eight *CaCOL* genes participated in the resistance to high-temperature adversity to varying degrees—five genes (*CaCOL01*, *CaCOL06*, *CaCOL07*, *CaCOL08*, and *CaCOL09*) were upregulated, and three genes (*CaCOL02*, *CaCOL03*, and *CaCOL05*) were downregulated. Most genes did not respond strongly to NaCl treatment, with some genes presenting a downward trend in expression, including *CaCOL02*, *CaCOL05*, and *CaCOL07*. Under osmotic stress, the expression levels of *CaCOL02*, *CaCOL03*, *CaCOL07*, and *CaCOL08* were upregulated after 2 h of treatment, while the other genes showed little or no change in expression (Figure 9).

## Discussion

To meet physiological needs and adapt to the external environment during the process of growth and development, plants usually control gene expression in vivo from multiple dimensions and levels, among which transcription level regulation is a crucial step (Jiao et al., 2007; Fornara et al., 2010). CO, located in the output pathway of the circadian clock, specifically activates the *FT* gene promoter to upregulate expression of the genes *FT* and *SUPPRESSOR OF OVEREXPRESSION OF CONSTANS 1* (*SOC1*), thus CO is an essential gene that regulates the photoperiod pathway and plays a central regulatory role (Yoo et al., 2005; Jiao et al., 2007; Tiwari et al., 2010). Members of the *COL* gene family have been identified in detail in the model plant *A. thaliana* and many other species (Griffiths et al., 2003; Song et al., 2015). *C. annuum* is an important vegetable crop, but there are limited studies on *COL* genes in this species. In the current study, 10 and 9 *COL* genes were identified in the genomes of two varieties—“*Zunla-1*” and “*CM334*”—of *C. annuum*, respectively, and these genes could be divided into three subgroups. *COL* genes of “*Zunla-1*” were distributed on five chromosomes, while those of “*CM334*” were distributed on six chromosomes. This may be due to chromosomal splicing or gene differentiation between varieties of *C. annuum*.

CONSTANS-like family members possess three conserved domains, two B-box domains and a CCT domain (Griffiths et al., 2003). B-box domains were markedly differentiated among the *CaCOL* genes, while the CCT domain was relatively more conserved. Phylogenetic analysis indicated that the B-box domain plays a crucial role in the phyletic evolution of *COL* gene family, which is consistent with previous studies (Griffiths et al., 2003; Song et al., 2015). The evolution of plant regulatory genes is relatively fast and the evolutionary rate is significantly different among different regions of COL family genes (Lagercrantz and Axelsson, 2000). Although both group I and group III COLs contain B-box domains, the domains of the two groups differ significantly. B-box domains of groups I and III were missing in some dicots (Song et al., 2015), but such loss was not observed in *C. annuum*. Compared with *A. thaliana*, the gene structures of *CaCOL* genes were relatively conserved in groups I and II, while those of group III showed apparent differentiation. Investigation of genetic diversity was critical in linking selection pressure to specific genes (Fu et al., 2010; Fiil et al., 2011; Wei et al., 2011). The lower the nucleotide diversity, the greater the selection pressure (Whitt et al., 2002). In the current study, COLs of 12 representative plants (*A. thaliana*, *B. rapa*, *C. Papaya*, *P. trichocarpa*, *F. vesca*, *V. vinifera*, *S. tuberosum*, *S. lycopersicum*, *C. annuum* cv. *Zunla-1*, *Z. mays*, *O. sativa*, and *A. trichopoda*) were analyzed to further explore the evolutionary history of the COLs. Genetic distance between *COL* genes was the biggest in group III and the smallest in group I, suggesting that *COL* genes in group III undergo more relaxed selection pressure during evolution compared with genes of the other two groups. The more extensive selection pressure and structural differences in group III may account for the functional differentiation of group III *COL* genes. Among the three groups of COLs, genetic distance was the smallest between groups I and III, and the largest between groups II and III, suggesting that groups II and III may be differentiated from group I (Figure 5B).

During their long evolutionary history, angiosperms have undergone different WGD events that induced gene duplication and differentiation (Taylor and Raes, 2004; Lee et al., 2012; Rensing, 2014). Due to the different regions and external environments of different plants, homologous genes are partially lost and partially retained during evolution. Retained genes undergo functional differentiation, leading to gradually different regulatory mechanisms (Wang et al., 2015). Analysis of the replication and retention of *COL* genes in the 12 selected plants demonstrated that *COL* genes had also undergone duplication, loss, retention, and differentiation during the evolutionary history of the plants, especially during the genome duplication event. Starting from the relict plant *A. trichopoda*, angiosperm genomes have undergone different WGD or WGT events (Clark and Donoghue, 2018), with the *COL* genes of groups I, II, and III presenting unequal amplification among the 12 angiosperm species. Compared with other angiosperms, group I *COL* genes of *C. annuum* had the highest proportion of retained genes, which was mainly caused by tandem replication. However, the tandem duplication genes (*CaCOL02*, *CaCOL03*, and *CaCOL04*) in group I were not significantly expressed in different tissues but did respond to abiotic stresses, thus they may exist as backup genes in response to other environmental factors, as indicated in a previous study (Qian et al., 2010; Duan et al., 2016). Among the other species, group III had the most retained genes, especially in *B. rapa*, which had up to 25 genes. Segmental duplications may be one of the reasons for this phenomenon (Song et al., 2015). Therefore, amplification of group III is presumed to be the main reason for the increase in the number of genes in the COL family. COLs play an essential regulatory role in the photoperiod pathway, but in the 12 selected species, which included typical long- and short-daylight plants, no obvious retention rule was found for *COL* genes. This indicated that photoperiod regulation had not affected the number of retained *COL* genes. Specific gene structures or functional differentiation of individual genes may account for their functions.

Obvious functional differentiation was also found in *COL* orthologous genes of the two varieties of *C. annuum*. Among these orthologous genes, four pairs had an identity up to 100%, while the remaining four pairs exhibited an average identity of 97.79%. The eight pairs of orthologous genes displayed similar expression trends in leaves, but in root tissue, only one gene was highly expressed in “*CM334*” and three genes were highly expressed in “*Zunla-1*.” Most of the *COL* orthologous genes in *C. annuum* and tomato had similar expression patterns in different tissues and developmental stages, but with considerable differences in some expression levels. These findings and analyses demonstrated that *COL* genes display great conservation but have also experienced loss and differentiation. This may be because different selection pressures in different environments result in different degrees of retention of *COL* genes, and during the retention process, some genes have undergone new functionalization or sub-functionalization.

COLs are instrumental in photoperiod regulation. Previous studies showed that transcription levels of COLs in tomato and *Arabidopsis* were regulated by circadian rhythms (Suarez-Lopez et al., 2001; Steinbach, 2019; Yang et al., 2020). In the current study, the expression patterns of *CaCOL* genes also exhibited a clear circadian rhythm under light induction. Under LD conditions, the expression trends of most homologous genes in pepper and tomato were similar, but they differed significantly from those in *Arabidopsis* (Suarez-Lopez et al., 2001; Yang et al., 2020). Expression patterns analysis showed that expression of *CaCOL01*, *CaCOL02*, *CaCOL03*, and *CaCOL08* peaked at 4 or 8 h after dawn, and their homologs in tomato had similar expression trends (Yang et al., 2020). However, *CaCOL01* and *CaCOL08*, as homologous genes of *AtCOL4*, were expressed at different levels in LD conditions. Additionally, the *CaCOL* genes showed different circadian rhythms (Figure 8), suggesting functional divergence of these genes in multiple aspects of plant development in response to the photoperiod. This may be because different species have different photoperiod requirements and that structurally similar genes have diverged functionally within or between species. In addition, COLs are also involved in the regulation of the development of different tissues, such as *GmCOL9* in soybeans and *MaCOL1* in bananas (Huang et al., 2011; Chen et al., 2012). In *C. annuum*, *CaCOL01*, *CaCOL06*, *CaCOL08*, and *CaCOL09* were highly expressed in stem and leaf. In addition, *CaCOL01*, *CaCOL08*, and *CaCOL07* were homologous to CO have higher expression in flower buds and flower tissues, and *CaCOL01*, *CaCOL06*, and *CaCOL09* were highly expressed during fruit development. These findings indicated that *CaCOL* genes play an important regulatory role in *C. annuum* growth and development.

Analysis of the promoter regions of the *CaCOL* genes revealed that the regions contained not only light response elements but also a variety of adversity stress response elements, suggesting *CaCOL* genes may be associated with resistance to abiotic stress. *COL* homologous genes, such as *MaCOL1* (Chen et al., 2012), *AtCOL4* (Min et al., 2015), OsCOL9 (Liu H. et al., 2016), *Ghd2* (Liu J. et al., 2016), and *BnCOL2* (Liu et al., 2020), were reported to be involved in adversity expression in other plant species. In pepper, five *CaCOL* genes (*CaCOL02*, *CaCOL03*, *CaCOL06*, *CaCOL07*, and *CaCOL08*) were expressed under the four adversity treatments (cold, heat, salt, and osmotic stress), with all five genes significantly expressed in the temperature treatments. *CaCOL02* and *CaCOL03* were upregulated in low temperatures and downregulated in high temperatures. A previous study showed that the CO-HOS1 module was crucial for fine-tuning of photoperiodic flowering under short-term temperature fluctuations (Jung et al., 2012). This may be one of the reasons why *COL* genes respond to temperature regulation. The *CaCOL* genes may help plants better adapt to the survival environment by changing the flowering time in response to adversity. Results from the current study provide a specific direction for studying stress-tolerance mechanisms in plants.

## Conclusion

This study investigated the evolutionary characteristics and expression function of *C. annuum COL* genes and included structural characteristics, replication types, evolutionary patterns, and expression analysis. Findings from this study lay the foundation for the in-depth understanding of the function of the *COL* gene family members in *C. annuum* and will facilitate research on the molecular response mechanism of these genes during growth and development.

## Materials and Methods

### Identification of CONSTANS-Like Gene Family in Pepper

Pepper (*C. annuum* cv. *Zunla-1* and *CM334*) genome data were downloaded from Solanaceae Genomics Network^1^ (Kim et al., 2014; Qin et al., 2014). To identify all COL family genes in the pepper genome, a batch Basic Local Alignment Search Tool (BLAST) search was performed against the pepper genome data using the full-length amino acid sequences of COs and COLs of *Arabidopsis* (Griffiths et al., 2003) as queries with an *E*-value cut-off of 1 × 10^––10^. All retrieved protein sequences were submitted to Pfam^2^ databases to annotate the protein domains. Only proteins containing the N-terminal B-box domain (PF00643.19) and C-terminal CCT domain (PF06203.9) were considered to be COL homologs in pepper. Candidate genes were verified in Simple Modular Architecture Research Tool (SMART) and National Center for Biotechnology Information (NCBI) databases to derive the final sequences.

Physical and chemical properties of the CaCOL proteins, including molecular weight (MV) and isoelectric point (pI), were determined and analyzed using EMBOSS programs^3^.

### Phylogenetic Tree, Gene Structure, and Conserved Domain Analysis of CONSTANS-Like Gene Family Members

Multiple sequence alignment analyses were performed using the CLUSTALW program (Thompson et al., 2002). Phylogenetic trees were then reconstructed by the Neighbor-Joining (NJ) method with a bootstrap value of 1000, using MEGA 6.0 software. The genetic distance of *COL* genes was also calculated using MEGA 6.0 software (Tamura et al., 2013).

Gene structure was plotted using the online tool Gene Structure Display Server (GSDS)^4^. Based on the *Arabidopsis thaliana* General Feature Format (GFF) file and *C. annuum* genome annotation information, the local Perl program was used to extract the location information of exons and introns and convert them into a GSDS readable bed. Online software MEME 4.9.0 was used to predict and analyze structural domains of COL protein sequences of *A. thaliana* and *C. annuum*; the motif search value was set to 10, the structural domain width was set to a minimum of 10 and a maximum of 100, and the other parameters used default settings. MEME structures were plotted using Tbtool software (Chen et al., 2020). The COL domain model was analyzed through the SMART database^5^.

### Chromosome Location and Orthologous and Paralogous Genes of the CaCOL Gene Family

Chromosome position information of *CaCOL* genes was obtained through the GFF file of annotation information of *the C. annuum* genome. The local Perl program was used to extract location information and construct the *C. annuum* chromosome map. The duplication type of *CaCOL* genes was analyzed by duplicate_gene_classifier, the downstream program of MCScanX (Wang et al., 2012).

OrthoMCL^6^ software was used to identify orthologous and paralogous *COL* genes of *C. annuum*, *S. lycopersicum*, and *A. thaliana* (Li et al., 2003). Orthologous and paralogous relationship diagrams of *COL* genes in the three genomes were plotted using Circos software^7^ (Krzywinski et al., 2009).

### Prediction of *Cis*-Elements in Promoters of CaCOL Genes

Based on the genome sequence of *C. annuum* cv. *Zunla-1* (Qin et al., 2014), the upstream 2-kb sequences of *CaCOL* genes were extracted using the local Perl program and submitted to the PlantCARE website^8^ for prediction of *cis*-acting elements. Finally, a *cis*-element distribution map was plotted by Tbtool software (Chen et al., 2020).

### Analysis of Expression of CaCOL Genes in Different Tissues

Transcriptome data from different tissues and fruit development stages of *C. annuum* cv. *Zunla-1*, *C. annuum* cv. *CM334*, and tomato were explored in this study (Tomato Genome Consortium, 2012; Kim et al., 2014; Qin et al., 2014). An expression heatmap was plotted using R software (R Core Team, 2013). Pearson correlation coefficient (PCC) values of the expression relationships among *CaCOL* genes in different tissues were calculated by the local Perl program, and the co-expression network was constructed with the Cytoscape 3.7 program (Shannon et al., 2003).

### Planting and Treating of Pepper

The pepper cultivar “Xianhong No. 1” was used for expression pattern analysis. Seeds were disinfected (2% sodium hypochlorite) and sown in the growth room of Huaiyin Institute of Technology, Jiangsu Province, China at 25/18°C day/night temperature, and with a 16/8-h light/dark cycle, relative humidity of 60–70%, and light intensity of 6000 Lux. Various treatments were applied to 45-day old seedlings and samples for RNA extraction were collected from leaves. Consistently robust 45-day-old plants were selected for 24 h sampling, collecting leaf tissues from the same leaf position at an interval of 4 h and performing three biological replicates. For abiotic stress treatments, the seedlings were placed over a plastic box filled with Japanese garden test nutrient solution, pH 6.0. Salt stress was applied by adding NaCl to a final concentration of 200 mmol/L in the nutrient solution; osmotic stress was imitated by adding polyethylene glycol 6000 (PEG6000) to a final concentration of 20%; cold or heat stress treatments were applied by transferring the seedlings into a growth chamber at 10 or 42°C, respectively. The illumination, photoperiod, and relative humidity of the treated plants were kept the same as the plants without abiotic stress treatments. A parallel control (normal growth) was set for each treatment. The second true leaves were sampled at 0, 1, 2, 4, 6, 12, and 24 h after treatment, and three biological replicates were performed at each time point. All samples were wrapped in tin foil, quickly placed in liquid nitrogen, and then stored in a refrigerator at –80°C.

### RNA Extraction and qRT-PCR Expression Analysis

A Simple Total RNA Kit (TIANGEN) was used to extract high-quality RNA from pepper leaves, and cDNA was derived by reverse transcription using a PrimeScript RT reagent kit (TaKaRa kit). Actin (GenBank accession number GQ339766.1) was used as an internal reference gene. qRT-PCR was performed (three technical repeats) according to the instructions of the SYBR premix Ex Taq kit (TaKaRa). The 2^–ΔΔ*CT*^ method was used to calculate the relative expression of *CaCOL* genes (Heid et al., 1996). Primers used for the expression analysis are listed in Supplementary Table 4.

## Data Availability Statement

The original contributions presented in the study are included in the article/Supplementary Material, further inquiries can be directed to the corresponding author/s.

## Author Contributions

WD and ZH conceived the study and collected the public dataset of the researched species. XB and ZH contributed to data analysis, bioinformatics analysis, and manuscript preparation. GC, BX, and RC participated in the qRT-PCR experiment. All authors read and approved the final version of the manuscript.

## Conflict of Interest

The authors declare that the research was conducted in the absence of any commercial or financial relationships that could be construed as a potential conflict of interest.

## Publisher’s Note

All claims expressed in this article are solely those of the authors and do not necessarily represent those of their affiliated organizations, or those of the publisher, the editors and the reviewers. Any product that may be evaluated in this article, or claim that may be made by its manufacturer, is not guaranteed or endorsed by the publisher.

## Footnotes

1
https://solgenomics.net/organism/Capsicum_annuum/genome

2
http://pfam.xfam.org/

3
https://www.ebi.ac.uk/Tools/emboss/

4
http://gsds.cbi.pku.edu.cn

5
http://smart.embl-heidelberg.de

6
http://orthomcl.org/orthomcl/

7
http://circos.ca/

8
http://bioinformatics.psb.ugent.be/webtools/plantcare/html/
