# Supplementary material for: Genome-Wide Identification and Expression Profiling of CONSTANS-Like Genes in Pepper (Capsicum annuum): Gaining an Insight to Their Phylogenetic Evolution and Stress-Specific Roles
Source: Front Plant Sci. 2022 Feb 17;13:828209. doi: 10.3389/fpls.2022.828209 (PMC8892298; doi:10.3389/fpls.2022.828209)
Supplement: Supplementary file 1 [file Data_Sheet_1.PDF]

Supplementary Material

Genome-wide identification and expression profiling of CONSTANS-like (COL) genes in pepper (*Capsicum annuum*): Gaining an insight to their phylogenetic evolution and stress-specific roles

Zhinan Huang[1#], Xueying Bai[1#], Weike Duan[1]*, Boqing Chen[1], Guodong Chen[1], Binghua Xu[2], Rui Cheng[2], Jizhong Wang[1]

[1] College of Life Sciences and Food Engineering, Huaiyin Institute of Technology, Huai'an 223003, P.R. China

[2] Huai'an Key Laboratory for Facility Vegetables, Huaiyin Institute of Agricultural Sciences of Xuhuai Region of Jiangsu, Huai'an, 223001, China

[#]These authors contributed equally to this work.

*** Correspondence:**
Weikeduan
weikeduan@126.com

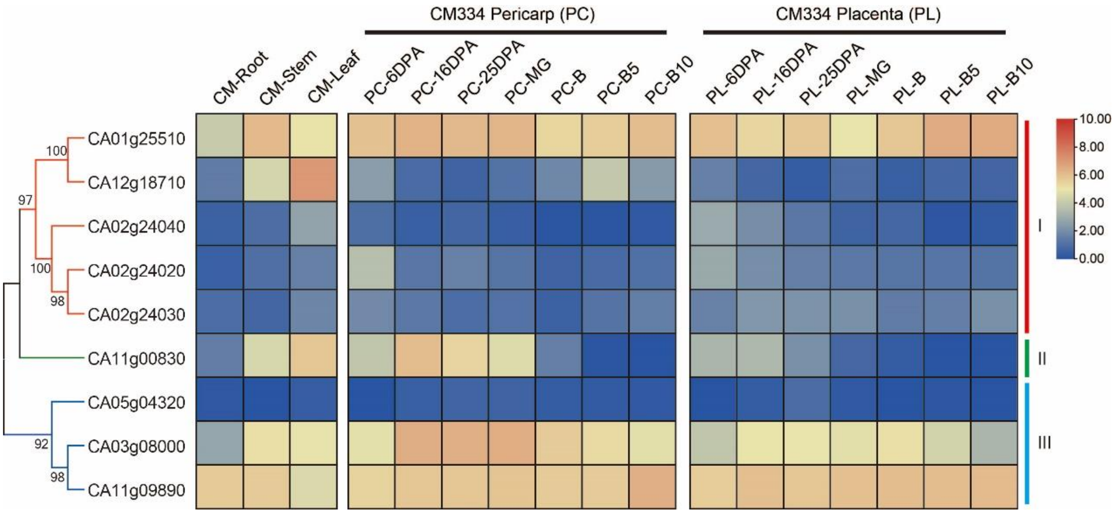

Figure S1. Expression patterns of *C. annuum* cv. *CM334 COL* genes in different tissues and fruit development process.

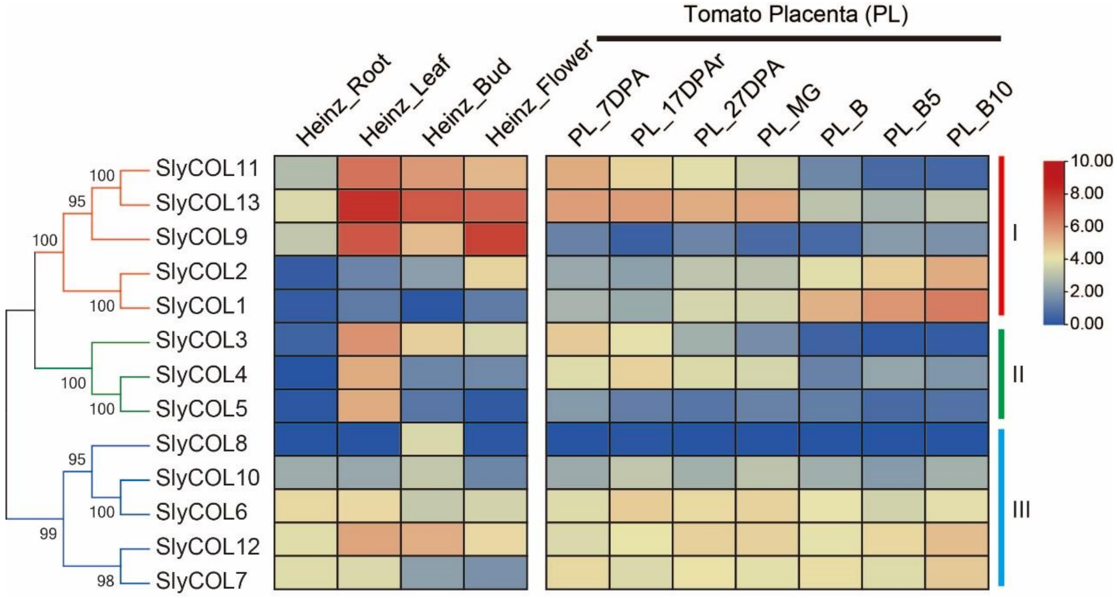

Figure S2. Expression patterns of tomato *COL* genes in different tissues and fruit development process.

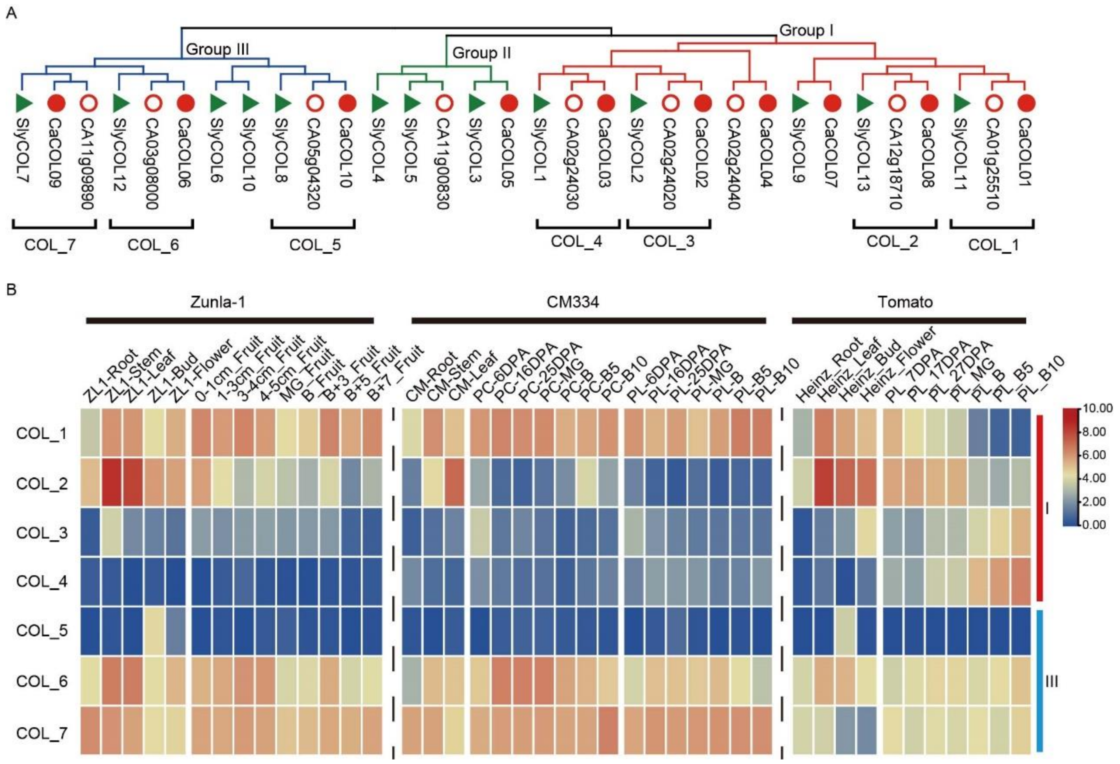

Figure S3. Phylogenetic relationship of COL gene family in *Capsicum annuum*, *Solanum lycopersicum* (A) and the expression patterns among the *COL* homologous genes (B). COLs of *C. annuum* cv. *Zunla-1*, *C. annuum* cv. *CM334* and tomato are indicated by red balls, red circle and green triangle, respectively. COL_1 to COL_8 represents the homologous gene pairs.
